# Metabolic responses to drought stress in the tissues of drought-tolerant and drought-sensitive wheat genotype seedlings

**DOI:** 10.1093/aobpla/ply016

**Published:** 2018-03-01

**Authors:** Rui Guo, LianXuan Shi, Yang Jiao, MingXia Li, XiuLi Zhong, FengXue Gu, Qi Liu, Xu Xia, HaoRu Li

**Affiliations:** 1 Institute of Environment and Sustainable Development in Agriculture (IEDA), Chinese Academy of Agricultural Sciences (CAAS)/Key Laboratory of Dryland Agriculture, Ministry of Agriculture, Beijing, P.R. China; 2 School of Life Sciences, Northeast Normal University, Changchun, China

**Keywords:** Drought stress, growth, metabolites, photosynthesis indices, wheat

## Abstract

An in-depth understanding of the effects of drought stress on plant metabolism is necessary to improve the drought tolerance of wheat and to utilize genetic resources for the development of drought stress-tolerant wheat varieties. In this study, the profiles of 58 key metabolites produced by wheat seedlings in response to drought stress were investigated to determine various physiological processes related to drought tolerance between drought-tolerant and drought-sensitive wheat genotypes. Results showed that the wheat metabolome was dominated by sugars, organic acids and amino acids; the wheat metabolome played important roles to enhance the drought tolerance of shoots. Under drought stress, JD17 exhibited higher growth indices and higher photosynthesis ability than JD8. A high level of compatible solutes and energy in shoots were essential for wheat to develop drought tolerance. Drought also caused system alterations in widespread metabolic networks involving transamination, tricarboxylic acid cycle, glycolysis, glutamate-mediated proline biosynthesis, shikimate-mediated secondary metabolisms and γ-aminobutyric acid metabolisms. Long-term drought stress resulted in the drought-tolerant wheat genotype JD17, which induced metabolic shifts in the tricarboxylic acid cycle and glycolysis with the depletion of the γ-aminobutyric acid shut process. In JD17, the prolonged drought stress induced a progressive accumulation of osmolytes, including proline, sucrose, fructose, mannose and malic acid. This research extended our understanding of the mechanisms involved in wheat seedling drought tolerance; this study also demonstrated that gas chromatography–mass spectrometry metabolomics could be an effective approach to understand the drought effects on plant biochemistry.

## Teaser

Productivity of wheat is affected by drought stress; however, the correlation between drought stress and metabolism is still not clear. The aim of this study was to investigate physiological adaptive mechanisms and reveal physiological processes related to drought tolerance. The results indicated that the wheat metabolome is dominated by sugars, organic acids and amino acids; the wheat metabolome plays important roles in enhancing the drought tolerance of shoots. Drought caused system alterations in widespread metabolic networks involving transamination, tricarboxylic acid cycle, glycolysis, glutamate-mediated proline biosynthesis, shikimate-mediated secondary metabolisms and GABA metabolisms.

## Introduction

Drought has affected humans since the emergence of agriculture and has caused the collapse of several civilizations (Stendle and Peterson 1998; Zhao *et al.* 2009). Drought remains prevalent in the modern era; for instance, drought affected 1.13 × 10^7^ hm^2^ of agricultural land in China in the 1970s and doubled to 2.667 × 10^7^ hm^2^ in the 1990s. The effect of drought has been countered by developing water-saving agricultural practices based on engineering, agronomy and water management (Wang *et al.* 2002; Luis *et al.* 2012). Biotechnology is in its infancy with regard to accelerating production of drought-tolerant crops (Shi 1999; Shao *et al.* 2007; Plauborg *et al.* 2010). However, progress in this area is significantly hampered by the physiological and genetic complexity of the drought tolerance trait. Thus, an enhanced understanding of drought tolerance mechanisms is necessary to improve crop varieties.

Drought is caused by insufficient water for uptake; this phenomenon inhibits further nutrient absorption and affects crop growth, gene expression, distribution, yield and quality (Stendle and Peterson 1998; Zhao *et al.* 2009). To tolerate drought stress, plants have evolved adaptive mechanisms, including accumulation of high concentrations of compatible solutes in the cytoplasm to counteract drought stress (Egilla *et al.* 2001; Chemikosova *et al.* 2006). Plant responses to drought stress may involve metabolic pathways, such as photosynthesis, sugar synthesis, tricarboxylic acid cycle, glycolysis and hormone synthesis (Spickett *et al.* 1992; Hare *et al.* 1998; Dennison *et al.* 2001). Metabolomic solutes, such as proline, betaine, fructose and sucrose, contribute to drought stress tolerance (Chen and Murata 2002; Yasar *et al.* 2006; Wang *et al.* 2012). Metabolomic components may also participate in plant drought tolerance; however, information regarding drought tolerance-related metabolomic components is limited. A comparative metabolic analysis of the responses of drought-tolerant genotypes and drought-sensitive genotypes to drought stress should be conducted to determine the mechanisms related to drought stress adaptation and to understand plant drought tolerance (Abebe *et al.* 2003; Miller *et al.* 2010). Metabolomic analyses have been applied to examine the abiotic stress tolerance of plants; these analyses can determine the specific responses of biological systems to genetic and environmental changes (Renberg *et al.* 2010; Oliver *et al.* 2011). Metabolomic analyses include various approaches, such as metabolic fingerprinting, metabolite profiling and targeted analysis, gas chromatography–mass spectrometry (GC–MS), liquid chromatography–Fourier transform mass spectrometry and nuclear magnetic resonance, to identify small-molecule metabolomic components. These technologies can be employed to identify metabolomic components accurately (Meng *et al*. 2011; Ruan *et al.* 2011; Barding *et al.* 2013). In wheat (*Triticum aestivum*), the metabolite profiling in response to salt stress (Wu *et al.* 2013), temperature (Kobayashi *et al.* 2004), N nutrition (Allwood *et al.* 2015) and drought stress has been analyzed (Bowne *et al.* 2012). However, research on metabolomics has yet to be conducted to investigate the physiological and molecular differences in drought tolerance between drought-tolerant and drought-sensitive wheat genotypes.

In this study, drought-tolerant wheat genotypes and drought-sensitive wheat genotypes were selected and utilized to compare growth, photosynthetic indices and metabolic changes in the genotypes in response to drought stress in tissues through GC–MS. This study aimed to define the possible metabolomic profiles of wheat plants and to determine the physiological adaptive mechanisms by which wheat tolerates drought stress.

## Methods

### Plant materials and cultivation

The seeds of drought-tolerant wheat genotype (JingDong-17) and drought-sensitive wheat genotype (JingDong-8) were disinfected with 3% H_2_O_2_ for 20 min, rinsed with distilled water and soaked for 12 h at room temperature. The seeds were sown in 17 cm diameter plastic pots (20 seeds per pot), and each wheat genotype was planted on 25 pots. Each pot contained 2.5 kg of washed sand. The seedlings were watered daily with half-strength Hoagland’s nutrient solution. All of the pots were placed outdoors but were sheltered from the rain; as a result, the day/night temperature range was 21.0–25.5 °C/18.5–21.0 °C.

### Treatment and sampling

Twenty-five pots with wheat genotype seedlings growing uniformly were selected and divided randomly into five sets when the seedlings were 4 weeks old. Each set comprised five pots. Each pot was considered as one replicate with five replicates per set. One set was used to determine the growth index at the beginning of treatment, two sets were utilized as the untreated control group and the last two sets were considered as the stress treatment group. The pots subjected to drought stress treatments were not watered for 15 days; the control plants were watered daily. Wheat seedlings were harvested 15 days after drought treatment and before seedling death. After 15 days, one set of the control group and one set of the stress treatment group samplings were frozen immediately in liquid nitrogen and stored at −80 °C to extract the metabolites. The last set of the two groups of samples were dried at 80 °C for 72 h, and the dry weight (DW) was recorded. Before the plants were harvested, the shoot length and the photosynthetic indices were measured and obtained.

### Measurement of growth

Relative growth rate (RGR) is defined as [ln DW at the end of drought stress treatment − ln DW at the start of stress treatment]/total treatment duration (Kingsbury and Epstein 1984). The absolute water content (AWC) of the seedlings was calculated as: (FW – DW)/DW, where FW is fresh weight (Yang *et al.* 2009).

### Measurement of photosynthesis indices

The photosynthetic indices were determined at 10:00 from the first fully expanded leaf blades by using an LI-6400XT portable open flow gas exchange system (Li-Cor, USA). The plants were treated with photosynthetically active radiation (PAR) of 1000 μmol m^−2^ s^−1^ (saturation irradiance) by utilizing red–blue light-emitting diodes. The maximum *PS*II quantum yield (*PS*II) was determined between 09:00 and 11:00 from fully expanded shoots by using Imaging-PAM (Walz, Effeltrich, Germany) (Genty *et al.* 1989). The shoots were stored in the dark for approximately 20 min before measurements were done. The intensities of the actinic and saturating light settings were 185 and 2500 μmol m^–2^ s^–1^ PAR, respectively. Randomly selected 500 mg aliquots of fresh shoots were extracted in acetone and evaluated to determine the content of carotenoids (*Car*) and chlorophylls (*Chl*) *a* and *b*. Each extract was analyzed thrice through spectrophotometry at 440, 645 and 663 nm. Calculations were based on the equations reported by Arnon (1949).

### Measurement of metabolites

Shoot extracts were prepared through the following procedures: approximately 100 mg of each frozen tissue sample was transferred into 2 mL centrifuge tubes, and 60 μL of water containing ribitol as an internal standard was added to each tube. After the mixtures were vortexed with 0.3 mL of methanol and 0.1 mL of chloroform, a 70 Hz grinding mill system (Jinxin Biotech Ltd, Shanghai, China) was utilized to grind the samples for 5 min. The samples were incubated at 70 °C for 10 min. The tubes were centrifuged at 12000 r.p.m. at 4 °C for 10 min. Supernatant (0.35 mL) was decanted into a 2 mL volume screw-top glass tube. The samples were dried in a vacuum concentrator at 30 °C for 2 h. Each sample was dissolved in 80 μL of methoxamine hydrochloride and incubated at 37 °C for 2 h. The samples were further derivatized with *N*,*O*-bis(trimethylsilyl)-trifluoroacetamide (BSTFA) containing 1% trimethylchlorosilane (100 μL) at 70 °C for 1 h. Gas chromatography–time-of-flight/mass spectrometry analysis was performed using a 1D Agilent 7890 gas chromatograph system coupled with a Pegasus 4D time-of-flight mass spectrometer. The system was equipped with a DB-5MS capillary column coated in 5% diphenyl cross-linked with 95% dimethylpolysiloxane (30 m × 250 μm inner diameter and 0.25 μm film thickness; J&W Scientific, Folsom, CA, USA). An aliquot of the analyte (1 μL) was injected in a splitless mode. Helium was adopted as carrier gas; the front inlet purge flow was 3 mL min^−1^; and the gas flow rate through the column was 1 mL min^−1^. The initial temperature was maintained at 90 °C for 0.25 min; temperature was increased to 180 °C at a rate of 10 °C min^−1^ and to 240 °C at a rate of 5 °C min^−1^. The temperature was further increased to 285 °C at a rate of 20 °C min^−1^ for 11.5 min. Injection, transfer line and ion source temperatures were 280, 270 and 220 °C, respectively. The energy was set at −70 eV in an electron impact mode. MS data were acquired in a full-scan mode with an *m*/*z* range of 20–600 at a rate of 100 spectra/s after a solvent delay of 492 s.

### Statistical analysis

Growth and photosynthetic activity variance and correlation were statistically analyzed using SPSS 13.0. All of the treatments were replicated five times. The means and calculated standard errors were reported. Metabolites were identified by searching FiehnLib, a commercial EI-MS library (Kind *et al.* 2009). The resulting 3D data, including peak number, sample name and normalized peak area, were run in SIMCA 14.0 software package (Umetrics, Umea, Sweden) and subjected to principal component analysis (PCA) and orthogonal projections to latent structure-discriminant analysis. Non-commercial databases, including KEGG (http://www.genome.jp/kegg/), were utilized to identify metabolite pathways. The format data were uploaded to the MetaboAnalyst website (www.metaboanalyst.ca/) for further analysis (Xia *et al.* 2012).

## Results

### Effect of drought stress on wheat seedling growth

**Figure 1.**
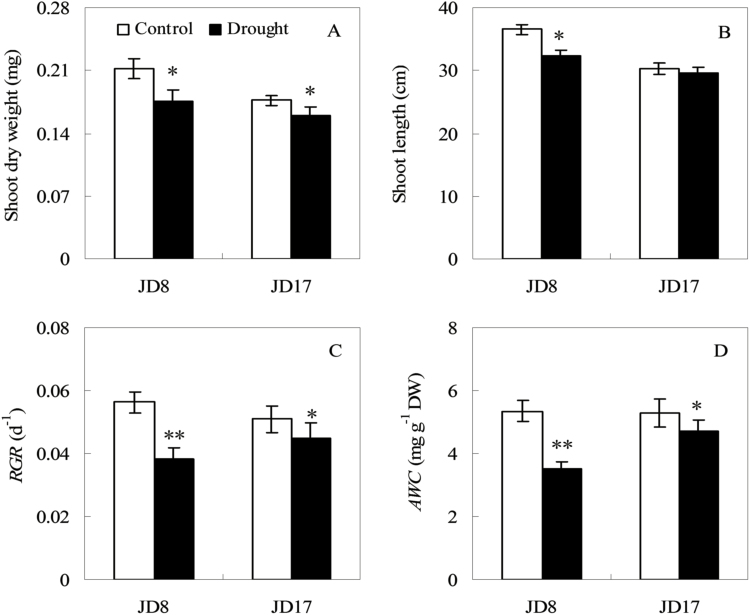
Growth performances of two wheat genotypes under control and drought stress conditions at the seedlings stage. (A) The shoot dry weight under control for 15 days of drought stress conditions; (B) Shoot length; (C) Shoot relative growth rate (*RGR*); (D) Absolute water content (*AWC*). Asterisk and double asterisk indicate significant (*P* < 0.05) and highly significant (*P* < 0.01) differences between controls and treatments, respectively.

Evident genotypic difference in growth was observed between the two genotypes after the drought treatment was administered (Fig. 1). The shoot dry weight and lengths of shoots were significantly affected by drought stress, which reduced the shoot dry weight at 16.9 and 9.5% in JD8 and JD17, and relative shoot length at 11.6 and 2.5%, respectively (Fig. 1A and B, *P* < 0.05). Only the lengths of the shoots of JD17 were not significantly different between the control treatment and the drought stress treatment (Fig. 1B). The RGR and AWC of the two different wheat genotypes decreased by 31.9 and 34.6% in JD8 (*P* < 0.01), whereas these parameters decreased by 11.8 and 10.5% in JD17 under drought stress, respectively (Fig. 1C and D, *P* < 0.05).

### Effect of drought stress on photosynthetic activity

**Figure 2.**
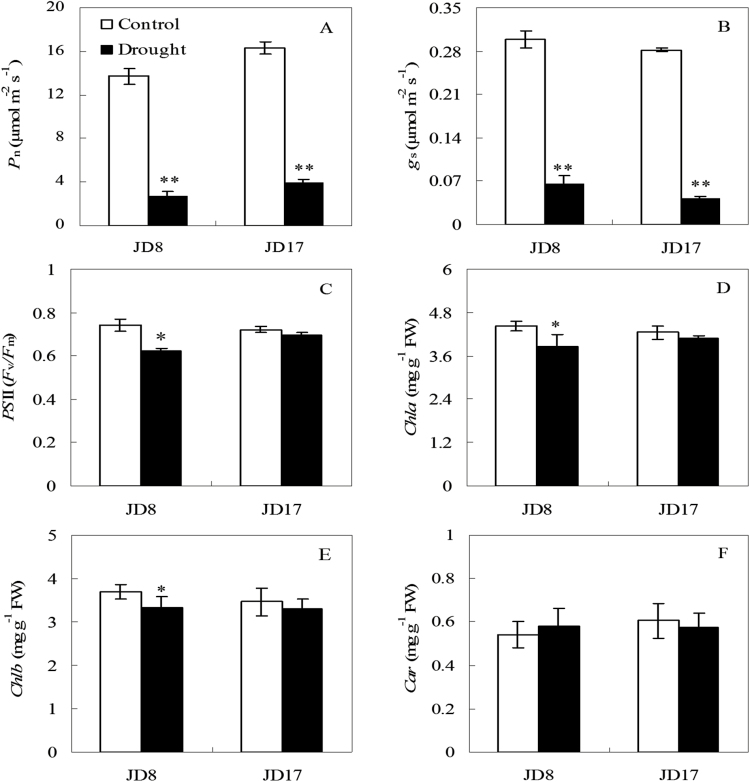
Photosynthetic activity of two wheat genotypes under control and drought stress conditions at the seedlings stage. (A) Net photosynthetic rate (*P*_n_) under control and for 15 days of no water irrigating conditions; (B) Stomatal conductance (*g*_s_); (C) The maximum quantum efficiency of PSII primary photochemistry (*PSII*); (D) Chlorophyll a (*Chla*); (E) Chlorophyll b (*Chlb*); (F) Carotenoid (*Car*). Asterisk and double asterisk indicate significant (*P* < 0.05) and highly significant (*P* < 0.01) differences between controls and treatments, respectively.

To determine the effect of drought stress on the photosynthetic activity at the seedling stage, the values of net photosynthetic rate (*P*_*n*_), stomatal conductance (*g*_*s*_), maximal PS II quantum yield (*PS*II) and pigments in shoots were identified. After 15 days of drought stress, both wheat genotypes showed highly significantly reductions in *P*_*n*_ and *g*_*s*_ compared with that of the corresponding controls (Fig. 2A and B, *P* < 0.01). The *PS*II value of JD8 was remarkably decreased under drought (Fig. 2C, *P* < 0.05). However, JD17 exhibited no significant difference compared with that of the control (Fig. 2C, *P* < 0.05). The altered trend of *Chl a* and *Chl b* were similar as *PS*II. The contents of *Chl a* and *Chl b* were significantly reduced by 12.4 and 10.1 of JD8 (Fig. 2D and E, *P* < 0.05). No significant difference in *Car* content was detected between the sample under drought stress and the control group in the shoots of JD8 and JD17 (Fig. 2F, *P* < 0.05).

### Metabolic changes in response to drought stress

**Figure 3.**
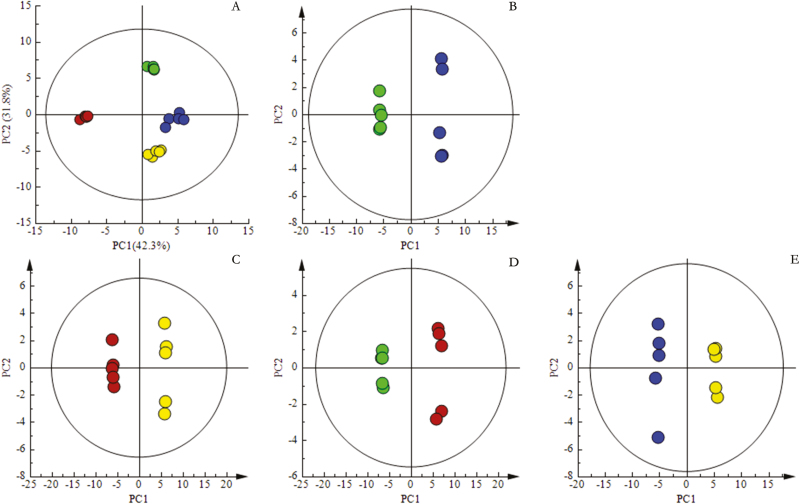
Principal component analysis (PCA) and orthogonal partial least squares discriminant analysis (OPLS-DA) of metabolic profiles in shoots of JD8 and JD17 under control and drought stress (five biological replicates). (A) PCA in shoots; (B) OPLS-DA between JD8-CK and JD8-DS; (C) OPLS-DA between JD17-CK and JD17-DS; (D) OPLS-DA between JD8-CK and JD17-CK; (E) OPLS-DA between JD8-DS and JD17-DS. Control, CK; Drought Stress, DS. JD8-CK is indicated in green; JD8-DS is indicated in blue; JD17-CK is indicated in red; JD17-DS is indicated in yellow.

To determine the physiological mechanisms of drought tolerance, the metabolic changes in the shoots responding to high drought were compared with those under normal growth conditions. A total of 58 types of metabolites were identified, and their corresponding concentrations were determined. PCA and OPLS-DA results (Figs 2 and 3) demonstrate an obvious distinction between samples under normal conditions and those subjected to drought treatment. The first principal component (PC1) and second principal component (PC2) represent 42.3 and 31.8% of the PCA, respectively (Fig. 3A). The contribution of metabolites in the shoots for PC1 was dominated by oxalic acid, galactose and succinic acid, whereas chiorogenic acid, cellobiose and aconitic acid were major contributors of PC2 (Table 1). Pairwise comparative OPLS-DA was carried out with one orthogonal and one predictive component calculated for all of the models derived from the two classes of samples to obtain detailed information on the metabolic alterations of JD8 and JD17 under control and drought stress and the significance of metabolites contributing to the alterations. In this research, OPLS-DA models determined the variation between samples within the control and drought treatments. The score plots of OPLS-DA results demonstrated evident variation between two wheat genotypes under control and drought stress with good model quality (Fig. 3B–E).

**Table 1.** Relative concentration and fold changes of metabolites in the shoots of JD8 and JD17 after 15 days of drought stress treatment. The relative concentration of each metabolite is an average of data from five biological replicates obtained through GC–MS. Fold changes were calculated using the formula Log_2_^(JD17/JD8)^ and log_2_^(drought/control)^. *P < 0.05.

Metabolites pathways	Metabolites name	Relative concentration				Fold changes			
		JD8		JD17		Log_2_^(JD17/JD8)^		Log_2_^(drought/control)^	
		CK	DS	CK	DS	CK	DS	JD8	JD17
*TCA cycle*	Citric acid	8.16 ± 0.89	1.60 ± 0.14	4.63 ± 0.29	2.94 ± 0.51	−0.82	0.87	−2.35*	−0.66
	Aconitic acid	40.97 ± 4.34	17.81 ± 1.70	16.31 ± 1.28	13.59 ± 1.35	−1.33*	−0.39	−1.20*	−0.26
	Isocitric acid	0.01 ± 0.00	0.02 ± 0.00	0.01 ± 0.00	0.02 ± 0.00	0.10	0.11	0.73	0.74
	α-Ketoglutaric acid	0.13 ± 0.02	0.16 ± 0.04	0.07 ± 0.01	0.10 ± 0.01	−0.95	−0.64	0.28	0.59
	Succinic acid	14.21 ± 1.55	15.91 ± 1.77	5.98 ± 0.18	13.78 ± 1.22	−1.25*	−0.21	0.16	1.20*
	Fumaric acid	0.04 ± 0.00	0.30 ± 0.03	0.01 ± 0.00	0.18 ± 0.01	−1.69*	−0.76	3.09*	4.02*
	Malic acid	10.84 ± 1.44	40.67 ± 4.31	12.04 ± 2.06	46.82 ± 4.18	0.15	0.20	1.91*	1.96*
	Oxalic acid	4.26 ± 0.51	6.59 ± 0.86	1.79 ± 0.48	4.33 ± 0.66	−1.25*	−0.60	0.63	1.28*
*Glycolysis*	Pyruvic acid	0.48 ± 0.11	0.35 ± 0.05	0.23 ± 0.03	0.47 ± 0.04	−1.03*	0.40	−0.43	0.99*
	Phenylpyruvate	0.00 ± 0.00	0.00 ± 0.00	0.00 ± 0.00	0.00 ± 0.00	−1.56*	0.57	0.64	2.77*
	Fructose-6-phosphate	0.14 ± 0.01	0.16 ± 0.03	0.35 ± 0.04	0.85 ± 0.02	1.29*	2.45*	0.11	1.27*
	Glucose-6-phosphate	0.21 ± 0.02	0.39 ± 0.03	0.14 ± 0.02	0.29 ± 0.01	−0.65	−0.41	0.87	1.11*
	Glucose	0.02 ± 0.00	0.04 ± 0.00	0.01 ± 0.00	0.05 ± 0.01	−1.20*	0.26	0.75	2.21*
	Sucrose	12.15 ± 0.98	8.12 ± 0.91	5.51 ± 0.76	17.05 ± 1.09	−1.14*	1.07*	−0.58	1.63*
	Fructose	46.68 ± 4.04	24.02 ± 3.15	12.55 ± 2.11	26.11 ± 3.02	−1.89*	0.12	−0.96*	1.06*
*Amino acids*	Proline	11.48 ± 1.20	26.97 ± 2.88	9.34 ± 1.02	58.45 ± 4.49	−0.30	1.12*	1.23*	2.65*
	Aspartate	10.63 ± 2.21	8.88 ± 1.76	12.28 ± 2.38	6.10 ± 0.89	0.21	−0.54	−0.26	−1.01*
	Serine	7.84 ± 0.78	10.80 ± 1.11	4.33 ± 0.66	14.22 ± 1.80	−0.86	0.40	0.46	1.72*
	Valine	4.73 ± 0.51	9.39 ± 1.09	2.66 ± 0.34	15.93 ± 0.58	−0.83	0.76	0.99*	2.58*
	Alanine	3.71 ± 0.31	3.61 ± 0.29	2.50 ± 0.33	3.71 ± 0.12	−0.57	0.04	−0.04	0.57
	Glycine	2.10 ± 0.28	2.18 ± 0.33	0.72 ± 0.05	3.09 ± 0.05	−1.55*	0.50	0.06	2.11*
	Isoleucine	2.04 ± 0.25	4.92 ± 0.59	1.10 ± 0.11	8.67 ± 0.72	−0.88	0.82	1.27*	2.97*
	Phenylalanine	1.28 ± 0.16	1.38 ± 0.14	1.10 ± 0.13	1.51 ± 0.11	−0.21	0.13	0.11	0.45
	Threonine	1.25 ± 0.16	2.62 ± 0.04	0.76 ± 0.08	4.04 ± 0.55	−0.72	0.63	1.07*	2.42*
	Leucine	0.97 ± 0.02	2.35 ± 0.13	0.68 ± 0.10	2.20 ± 0.78	−0.51	−0.10	1.28*	1.69*
	Lysine	0.69 ± 0.41	0.55 ± 0.05	0.22 ± 0.02	0.48 ± 0.03	−1.68*	−0.21	−0.33	1.13*
	Methionine	0.68 ± 0.10	0.48 ± 0.02	0.27 ± 0.03	0.37 ± 0.04	−1.31*	−0.39	−0.49	0.43
	Glutamate	0.93 ± 0.02	0.43 ± 0.03	1.98 ± 0.13	0.49 ± 0.02	1.09*	0.19	−1.11*	−2.01*
	Glutamine	1.57 ± 0.13	0.35 ± 0.02	2.02 ± 0.18	0.26 ± 0.02	0.36	−0.43	−2.17*	−2.96*
	Ornithine	0.28 ± 0.02	0.23 ± 0.02	0.15 ± 0.01	0.17 ± 0.02	−0.90	−0.42	−0.31	0.18
	Asparagine	0.41 ± 0.03	0.15 ± 0.01	0.59 ± 0.01	0.14 ± 0.01	0.52	−0.17	−1.42*	−2.11*
	L-Cysteine	0.04 ± 0.00	0.06 ± 0.01	0.02 ± 0.00	0.05 ± 0.00	−0.75	−0.33	0.49	0.91
	Citrulline	0.10 ± 0.02	0.16 ± 0.00	0.09 ± 0.01	0.17 ± 0.01	−0.10	0.05	0.70	0.85
	Tryptophan	0.06 ± 0.00	0.05 ± 0.00	0.03 ± 0.00	0.05 ± 0.00	−1.16*	−0.06	−0.25	0.85
*Sugars and polyols*	Mannose	107.54 ± 9.76	23.03 ± 0.45	55.54 ± 3.68	156.74 ± 11.26	−0.95*	2.77*	−2.22*	1.50*
	myo-Inositol	63.37 ± 4.87	45.01 ± 5.12	35.48 ± 1.99	36.47 ± 1.57	−0.84	−0.30	−0.49	0.04
	Lyxose	39.38 ± 2.05	43.31 ± 1.98	18.34 ± 0.79	31.28 ± 1.66	−1.10*	−0.47	0.14	0.77
	Galactinol	13.40 ± 0.59	12.85 ± 1.22	7.03 ± 0.59	6.81 ± 0.62	−0.93	−0.92	−0.06	−0.05
	Tagatose	2.27 ± 0.04	1.76 ± 0.18	1.21 ± 0.12	2.61 ± 0.33	−0.91	0.57	−0.37	1.11*
	Altrose	1.44 ± 0.14	1.64 ± 1.36	0.93 ± 0.10	1.36 ± 0.28	−0.63	−0.27	0.18	0.55
	Glucoheptose	0.46 ± 0.30	0.42 ± 0.05	0.29 ± 0.02	0.46 ± 0.06	−0.69	0.12	−0.14	0.67
	Ethanolamine	0.75 ± 0.06	0.69 ± 0.07	0.41 ± 0.03	0.65 ± 0.03	−0.88	−0.10	−0.12	0.66
	Galactose	0.31 ± 0.03	0.34 ± 0.04	0.14 ± 0.01	0.35 ± 0.05	−1.20*	0.02	0.15	1.36*
	Lactose	0.21 ± 0.01	0.29 ± 0.02	0.10 ± 0.02	0.26 ± 0.01	−1.01*	−0.15	0.47	1.33*
	Fucose	0.12 ± 0.01	0.11 ± 0.01	0.05 ± 0.00	0.08 ± 0.00	−1.35*	−0.48	−0.11	0.76
	Gentiobiose	0.12 ± 0.01	0.22 ± 0.03	0.07 ± 0.00	0.20 ± 0.01	−0.79	−0.12	0.86	1.53*
	Xylose	0.09 ± 0.01	0.10 ± 0.00	0.03 ± 0.00	0.08 ± 0.00	−1.50*	−0.28	0.09	1.31*
	Cellobiose	0.07 ± 0.00	0.02 ± 0.00	0.01 ± 0.00	0.01 ± 0.00	−2.43*	−0.87	−1.55*	0.01
	Trehalose	0.03 ± 0.00	0.01 ± 0.00	0.02 ± 0.00	0.05 ± 0.00	−0.66	2.42*	−1.65*	1.43*
	Sedoheptulose	0.03 ± 0.00	0.05 ± 0.00	0.01 ± 0.00	0.03 ± 0.00	−1.18*	−0.77	0.76	1.17*
*GABA shut*	γ-Aminobutyric acid	33.31 ± 1.75	40.62 ± 2.84	36.02 ± 2.45	17.37 ± 0.77	0.11	−1.23*	0.29	−1.05*
	Putrescine	0.02 ± 0.00	0.01 ± 0.00	0.01 ± 0.00	0.01 ± 0.00	−0.89	−0.35	−1.55*	−0.62
	Succinate Semialdehyde	0.06 ± 0.00	0.10 ± 0.01	0.05 ± 0.00	0.10 ± 0.00	−0.25	0.04	0.67	0.96
*Shikimic path way*	Shikimic acid	17.89 ± 0.63	18.78 ± 1.17	5.40 ± 0.54	8.14 ± 0.50	−1.73*	−1.20*	0.07	0.59
	Quinic acid	4.09 ± 0.65	3.94 ± 0.29	1.35 ± 0.12	1.36 ± 0.10	−1.60*	−1.53*	−0.05	0.02
	Cinnamic acid	0.07 ± 0.01	0.03 ± 0.00	0.06 ± 0.00	0.05 ± 0.01	−0.20	0.87	−1.32*	−0.25
	Chlorogenic acid	0.53 ± 0.01	0.25 ± 0.02	0.22 ± 0.04	0.12 ± 0.01	−1.28*	−1.00*	−1.11*	−0.83
	Ferulic acid	0.06 ± 0.00	0.08 ± 0.00	0.05 ± 0.00	0.05 ± 0.00	−0.45	−0.65	0.28	0.08

### Difference in metabolic profiles between drought-tolerant genotype and drought-sensitive wheat genotype under normal condition or drought stress

Genotypic difference in metabolic profiles was observed under the normal condition. The drought-sensitive genotype JD8 exhibited dramatically higher contents of 24 metabolites and radically lower contents of two metabolites in shoots compared with that of the drought-tolerant genotype JD17 (Table 1, *P* < 0.05). These up-accumulated metabolites in JD8 were mainly sugars and organic acids, including cellobiose, fructose and sucrose, and shikimic acid, fumaric acid and quinic acid. In contrast, the fructose-6-P and glutamate contents of JD17 were significantly higher than that of JD8 (Table 1, *P* < 0.05).

In shoots, there were five and four metabolites showing significantly higher and lower contents in JD17 than those in JD8 under drought stress, respectively (Table 1, *P* < 0.05). The up-accumulation metabolites in JD17 included mannose, fructose-6-P, trehalose and proline (Table 1, *P* < 0.05). The difference in mannose content between the two genotypes was the most significant, implying the higher capability of the drought-tolerant genotype wheat in sugar biosynthesis and carbon storage in shoots. The metabolites that exhibited an increase in JD8 were quinic acid, glutamate, shikimic acid and chlorogenic acid (Table 1, *P* < 0.05).

## Discussion

### Effect of drought stress on wheat seedling growth and photosynthetic activity

During the seedling stage, the plants are sensitive to adverse external factors because the initial performance of plants significantly affects plant growth and development (Paz and Martinez-Ramos 2003; Du and Huang 2008). The results showed that the dry weight and lengths of shoots in JD8 were significantly reduced than that in JD17 under drought stress (Fig. 1A and B, *P* < 0.05). Relative growth rate and AWC reflect the life-sustaining activities of the plant and are considered the optimum indices for the degree of stress and response of plants to various stresses; thus, these indices should be considered in evaluating drought tolerance (Yang *et al.* 2007). In this study, the RGR and AWC of the two wheat genotypes were inhibited under drought stress. However, the reduction of JD8 was greater than that in JD17 (Fig. 1C and D, *P* < 0.05). The phenomenon implies that the decrease in RGR was caused by the decrease in *P*_*n*_. The results indicated that the mechanisms of drought tolerance in JD8 and JD17 differ, and the JD17 tends to maintain a relatively high growth under drought stress. The results were consistent with the findings in the literature (Sun *et al.* 2001; Zhao and Tian 2008; Shan *et al.* 2012).

The rate of plant photosynthesis usually decreases with increasing stress intensity (Koyro 2006; Wei *et al.* 2006). Drought stress remarkably influenced the indices of photosynthesis with *P*_*n*_ exhibiting a substantial decrease (Fig. 2A, *P* < 0.01). *g*_*s*_ was closely correlated with the change in wheat AWC. The change in *g*_*s*_ of wheat resulted from the response to the decrease in environmental water potential and intracellular AWC (Fig. 2B, *P* < 0.01). The reduction of wheat *P*_*n*_ is considered to be a result of the decrease of *g*_*s*_ caused by stomatal factors, which depend on the cumulative effects of shoot water and osmotic potential (Bethke and Drew 1992). The effect of the 15-day drought stress on the leaf fluorescence properties of different wheat genotypes demonstrates that *PS*II shows a different degree decrease. JD8 reduced significantly by 15.7%. However, JD17 did not achieve a significant level of reduction (Fig. 2C, *P* < 0.05). The results indicated that photoinhibition occurs and the photosynthetic tissue *PS*II of JD8 was destroyed under drought stress. However, this outcome had not occurred in JD17. *Chl* and *Car* are the main photosynthetic pigments in higher plants (Cartelat *et al.* 2005). Under drought stress, *Chl a* and *Chl b* of JD17 were stimulated, but these pigments decreased sharply in JD8 (Fig. 2C and D, *P* < 0.05). This result implies that drought stress may enhance the activity of the *Chl*-degrading enzyme chlorophyllase in JD8 (Shi and Zhao 1997; Yang *et al.* 2008). The results demonstrated that the chlorophyll content of shoots decreased rapidly with the increase of drought stress time, causing the transfer rate from *LHC*II to *PS*II to decrease and protein concentration of the complex to decline rapidly. Compared with the drought-sensitive wheat genotype, drought-tolerant wheat genotype exhibited a protection mechanism. These results are consistent with those obtained by Yang *et al.* (2004) and Tambussi *et al.* (2005).

### Metabolic changes in response to drought stress

Metabolome research in plant systems is progressing, and there are three different applications of metabolome analysis, including target metabolic analysis, metabolomic analysis and metabolic fingerprinting analysis (Bailey *et al.* 2003; Schaneberg *et al.* 2003; Verdonk *et al.* 2003). In this study, we used metabolomic analysis to study two wheat genotypes, conducting a metabolic pathway analysis and metabolic network analysis under the same drought stress conditions. Changes in the metabolism were relatively stable after 15 days drought stress treatment, which is a suitable time period for studying the relationship between metabolites and drought resistance of wheat.

**Figure 4.**
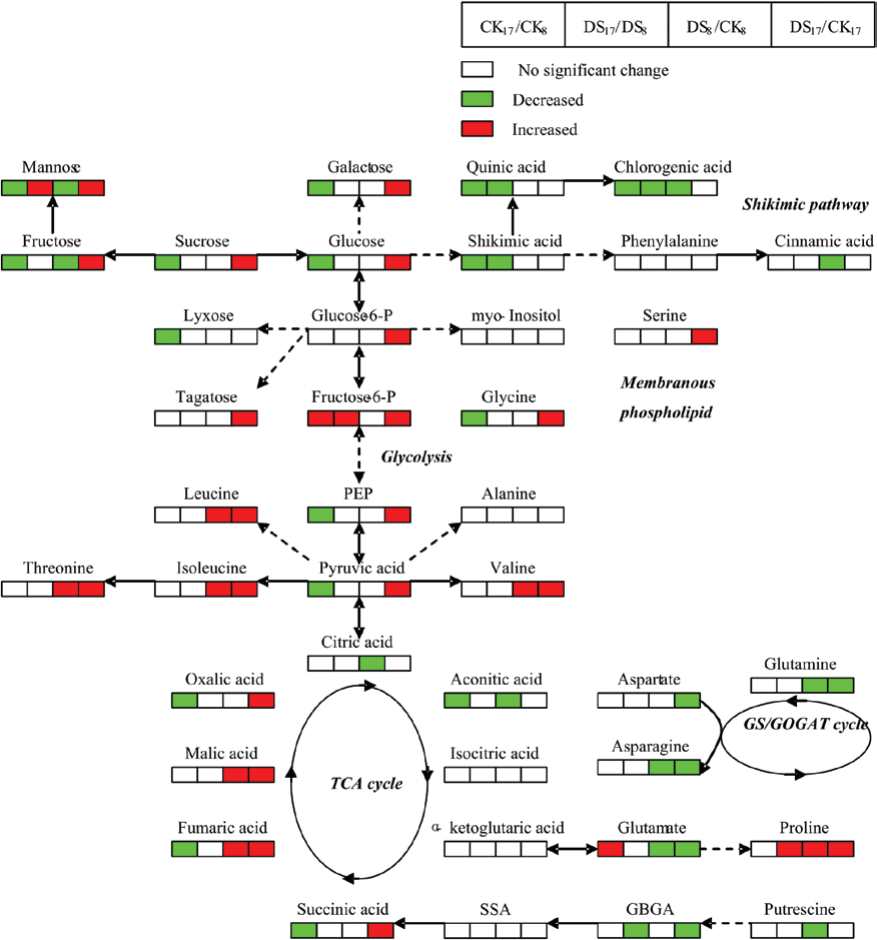
Change in metabolites of the metabolic pathways in shoots of JD8 and JD17 after 15 days of drought treatment, metabolic changes for wheat plants upon drought stress obtained from OPLS-DA analysis. The proposed metabolic pathways were based on web-based metabolic pathway database MetaCyc (http://www.metacyc.org) and the literature. Metabolites with red boxes denote significant increases while with green ones denote significant decreases. The bold-lettered metabolites were detected in this study. The level of significance was set at *P* < 0.05.

Based on the PCA results, the shoots under drought stress and those from the control differed in 19 and 32 metabolites with a significant change in JD8 and JD17, respectively (Table 1, *P* < 0.05). When compared with control samples, JD8 had significantly higher contents of 8 metabolites and significantly lower contents of 11 metabolites in shoot, meanwhile, 28 and 4 metabolites showed significantly higher and lower contents, respectively (Table 1, *P* < 0.05). Some metabolites exhibited a similar change in response to drought stress in both genotypes. Under drought stress, the metabolites that showed significant increase were fumaric acid, malic acid, proline, valine, isoleucine, threonine and leucine. In shoots, metabolites that substantially decreased included glutamate, glutamine and asparagine. Nevertheless, the magnitude of these changes was more evident in JD17 than JD8 (Table 1, *P* < 0.05). Meanwhile, in response to drought stress, the mannose, fructose, sucrose and trehalose content of shoots in JD17 increased significantly, whereas their content decreased in JD8 (Table 1, *P* < 0.05). Furthermore, some types of organic acids and sugars significantly decreased under drought stress in JD8, including aconitic acid, citric acid, cinnamic acid and chlorogenic acid, and fructose, mannose and cellobiose (Table 1 and Fig. 4, *P* < 0.05). Moreover, JD17 exhibited significantly higher contents of 28 metabolites, such as succinic acid, oxalic acid, aspartic acid and serine (Table 1 and Fig. 4, *P* < 0.05), under drought stress than that in the control group. This result probably assumed that drought-sensitive wheat genotype has a greater capacity in regulating drought stress than drought-tolerant wheat genotype by producing more sugars, organic acids and amino acids in shoots.

The response of metabolites to drought stress varied between the two wheat genotypes. TCA cycle was significantly enhanced in JD17 by succinic acid, fumaric acid, malic acid and oxalic acid, which were substantially increased, but not all in JD8 (Table 1 and Fig. 4, *P* < 0.05). In glycolysis, pyruvic acid, phenylpyruvate (PEP), fructose-6-P, glucose-6-P, glucose, sucrose and fructose were remarkably increased; this result indicated that sugar production was enhanced by drought stress in JD17 (Table 1 and Fig. 4, *P* < 0.05). In contrast, sucrose and fructose were significantly reduced, indicating that glycolysis was inhibited in JD8 (Table 1 and Fig. 4, *P* < 0.05). Both the γ-aminobutyric acid (GABA) shunt and shikimate pathway were inhibited under drought stress in D8, resulting in a decrease in putrescine, cinnamic acid and chlorogenic acid contents, whereas these acids were not significantly affected by drought stress in JD17 (Table 1 and Fig. 4, *P* < 0.05). The GABA shunt process of JD17 deceased under drought stress by the reduction of γ-aminobutyric acid levels, whereas shikimate pathway exhibited no significant change. Most amino acids in both wheat shoot genotypes increased under drought stress compared with those of the control treatment, although the extent of this increase was significantly higher in JD17 than that in JD8 (Table 1 and Fig. 4, *P* < 0.05).

Drought stress affects shoot functions upon exposure to drought stress; however, several organic molecules are known to play important roles during osmotic adjustment, including amino acids, sugars and organic acids, which potentially aid in balancing the osmotic potential of the vacuoles (Rhodes and Hanson 1993). The evident difference in the response of metabolites to drought stress between shoots, as well as genotypes, was observed. The results indicated that TCA cycle, glycolysis and sugar accumulation were enhanced; although the GABA shut process was inhibited in shoots under drought stress of drought-tolerant wheat genotypes (Table 1 and Fig. 4, *P* < 0.05). In contrast, TCA cycle, glycolysis and sugars synthesis appeared to be inhibited in shoots. Meanwhile, the GABA shut and shikimic path way were inhibited under drought stress in drought-sensitive wheat genotypes (Table 1 and Fig. 4, *P* < 0.05). The results implied that a high level of energy and sugar content is crucial for shoots to develop drought stress tolerance, and active synthesis metabolism is a basic response for shoots to tolerate drought stress (Santos and Pimentel 2009; Loutfy *et al.* 2012). The active synthesis metabolism of nutrients, including sugars, proline and organic acids, were dramatically enhanced in shoots, which improved the ROS detoxification capacity, osmotic adjustment, membrane stability and drought tolerance (Iqbal *et al.* 2011; Loutfy *et al.* 2012; Marcin′ska *et al.* 2013). On the basis of the comparison of metabolic profiles and the SPAD value between the two genotypes under control and drought stress, we may conclude that JD17 contains higher compatible solutes, exhibits a more active metabolite synthesis and shows a more rapid growth than JD8 under drought stress.

In response to drought stress, proline protects plant cell membranes and proteins and functions as a scavenger of reactive oxygen species (Delauney and Verma 1993; Hare *et al.* 1998). In the present work, proline levels increased by approximately 1.23-fold in the shoots of JD8, and by 2.65-fold in JD17. Furthermore, proline accounted for 22.7 and 35.6% of the free amino acids in the control group and up to 23.1 and 48.5% in JD8 and JD17 of those after drought stress exposure, respectively. The similar significant increase in proline contents was reported by Marcin′ska *et al.* (2013) and Chorfi and Taı¨bi (2011) in wheat. Proline accumulation is possibly caused by an increase in glutamate-mediated biosynthesis (Zhang *et al.* 2011). In this study, the decrease in transamination-related metabolites, including aspartate, glutamate, glutamine, asparagine and γ-aminobutyric acid, with prolonged drought stress is consistent with the diversion of metabolic activities to proline biosynthesis (Jander and Joshi 2010; Lehmann *et al.* 2010) (Table 1, *P* < 0.05). Furthermore, the decrease in the levels of these metabolites complement the demands of proline biosynthesis in JD17, and these demands were greater than those in JD8; this finding suggested that JD17 contains more excess metabolites that can be further converted into proline through the action of Δ1-pyrroline-5-carboxylate synthetase than JD8. The changes in valine, isoleucine, threonine and leucine are probably related to gluconeogenesis to relieve transamination products because these amino acids are glucogenic amino acids linked to pyruvate metabolism (Zhang *et al.* 2011). The increase in these amino acids was probably associated with the inhibition of protein biosynthesis or with enhanced protein degradation because plant growth was clearly inhibited with prolonged drought stress after 15 days. In JD17, the increase in serine and glycine derived from 3-phosphoglycerate is probably linked with glycolysis metabolism, which functions as plant endogenous antioxidants (Less and Galili 2008).

Studies have shown that sugars, including sucrose, fructose and glucose, are compatible solutes in response to drought stress (Ebstamp *et al.* 1994; Turk *et al.* 1999). Our results showed that the accumulation of some sugars, including sucrose, fructose, mannose and tagatose, in JD17 remarkably increased under drought stress (Table 1, *P* < 0.05). Changes in these sugars have been reported in drought-stressed cotton (*Gossypium hirsutum*) during long-term drought (Chang and Ryan 1987; Pettigrew 2004; Loka and Oosterhuis 2014). However, these sugars decreased in response to drought stress in sensitive genotype wheat JD8 (Table 1, *P* < 0.05). In this study, the accumulation of mannose increased with prolonged drought stress. Compared with that of the control, the mannose of the group exposed to drought stress accounted for 40.3–55.8% of the total sugars in JD17. Mannose was increased in the shoots of the drought-tolerant wheat genotype subjected to drought stress mainly because of the enhanced hexokinase (Kato *et al.* 2007; Wenzel *et al.* 2015). The two wheat genotypes accumulated malic acid, oxalic acid and fumaric acid to maintain intracellular ionic balance and nutrient uptake to resist drought stress (Table 1 and Fig. 4, *P* < 0.05). Malic acid exhibits a significant positive correlation with total organic acid content; the rate of malic acid utilization in sink tissues decreases under drought stress mainly because of the suppression of the NAD-dependent malate dehydrogenase (Rzepka *et al.* 2009). We suggest that the most important compatible solutes are proline, sugars (sucrose, fructose and mannose) and organic acids (malic acid, oxalic acid and succinic acid) in shoots.

## Conclusions

The comparison of metabolites in JD17 (drought-tolerant) and JD8 (drought-sensitive) wheat genotypes under control and drought stress conditions, showed that the levels of some metabolites differed between the two genotypes under drought stress. Compared with JD8, JD17 contained lower levels of fructose, sucrose and cellobiose in shoots and higher levels of fructose-6-P and glutamate under normal conditions. The results showed that JD8 exhibited higher capability of sugars synthesis than JD17. On the basis of the comparison results of metabolic profiles and SPAD values between drought-tolerant JD17 and drought-sensitive JD8 wheat genotype under drought stress, the harmful effects of drought stress on the distribution and accumulation of metabolites in JD8 were significantly greater than those in JD17. Under drought stress, JD17 accumulated higher levels of proline; sucrose, fructose, mannose and tagatose; malic acid, oxalic acid and fumaric acid in shoots. These metabolites are commonly considered as compatible solutes, which are involved in osmotic adjustment, protecting membranes and proteins from the damage by ROS. The results suggested that a high level of energy and sugar content is crucial for shoots to develop drought stress tolerance, and active synthesis metabolism is a basic response for shoots to tolerate drought stress. The active synthesis metabolisms of nutrients were dramatically enhanced in shoots, which improved the ROS detoxification capacity, osmotic adjustment, membrane stability and drought tolerance.

## Sources of Funding

This research was supported by grants from the Project of the National Natural Science Foundation of China (No. 31570328, 31200243), the National High-Tech R & D Program (863 Program) for the 12th Five-Year Plan (2011AA100503), the basic research special fund operations (No. BSRF201201) and National ‘Twelfth Five-Year’ Plan for Science & Technology Support (2011BAD09B01).

## Contributions by the Authors

R.G., L.X.S. and M.X.L. designed the research; R.G., L.X.S. and M.X.L. performed the research; R.G., L.X.S., M.X.L., Y.J., X.L.Z., F.X.G., Q.L., X.X. and H.R.L. analysed the data; and R.G., L.X.S., M.X.L., Y.J., X.L.Z., F.X.G. and Q.L. wrote the paper. All authors reviewed the manuscript.

## Conflict of Interest

None declared.

## References

[CIT0001] Abebe T, Guenzi AC, Martin B, Cushman JC 2003 Tolerance of mannitol-accumulating transgenic wheat to water stress and salinity. Plant Physiology 131:1748–1755. 12692333 10.1104/pp.102.003616 PMC166930

[CIT0002] Allwood JW, Chandra S, Xu Y, Dunn WB, Correa E, Hopkins L, Goodacre R, Tobin AK, Bowsher CG 2015 Profiling of spatial metabolite distributions in wheat leaves under normal and nitrate limiting conditions. Phytochemistry 115:99–111. 25680480 10.1016/j.phytochem.2015.01.007 PMC4518043

[CIT0003] Arnon DI 1949 Copper enzymes in isolated chlorop lasts phenoloxidases in *Beta vulgaris*. Plant Physiology 24:1–15. 16654194 10.1104/pp.24.1.1 PMC437905

[CIT0004] Bailey NJ, Oven M, Holmes E, Nicholson JK, Zenk MH 2003 Metabolomic analysis of the consequences of cadmium exposure in *Silene cucubalus* cell cultures via HNMR spectroscopy and chemometrics. Phytochemistry 62:851–858. 12590112 10.1016/s0031-9422(02)00719-7

[CIT0005] Barding GA Jr, Béni S, Fukao T, Bailey-Serres J, Larive CK 2013 Comparison of GC–MS and NMR for metabolite profiling of rice subjected to submergence stress. Journal of Proteome Research 12:898–909. 23205590 10.1021/pr300953k

[CIT0006] Bethke PC, Drew MC 1992 Stomatal and nonstomatal components to inhibition of photosynthesis in leaves of capsicum annuum during progressive exposure to NaCl salinity. Plant Physiology 99:219–226. 16668853 10.1104/pp.99.1.219 PMC1080428

[CIT0007] Bowne JB, Erwin TA, Juttner J, Schnurbusch T, Langridge P, Bacic A, Roessner U 2012 Drought responses of leaf tissues from wheat cultivars of differing drought tolerance at the metabolite level. Molecular Plant 5:418–429. 22207720 10.1093/mp/ssr114

[CIT0008] Cartelat A, Cerovic ZG, Goulas Y, Meyer S, Lelarge C, Prioul JL, Barbottin A, Jeuffroy MH, Gate P, Agati G, Moya I 2005 Optically assessed contents of leaf polyphenolics and chlorophyll as indicators of nitrogen deficiency in wheat (*Triticum aestivum* L.). Field Crop Research 9:35–49.

[CIT0009] Chang CW, Ryan RD 1987 Effects of water stress on starch and sucrose metabolism in cotton leaves. Starch 39:84–87.

[CIT0010] Chemikosova SB, Pavlencheva NV, Gur’yanov OP, Gorshkova TA 2006 The effect of soil drought on the phloem fiber development in long-fiber flax. Russian Journal of Plant Physiology 53:656–662.

[CIT0011] Chen TH, Murata N 2002 Enhancement of tolerance of abiotic stress by metabolic engineering of betaines and other compatible solutes. Current Opinion in Plant Biology 5:250–257. 11960744 10.1016/s1369-5266(02)00255-8

[CIT0012] Chorfi A, Taı¨bi K 2011 Biochemical screening for osmotic adjustment of wheat genotypes under drought stress. Tropicult 29:82–87.

[CIT0013] Delauney AJ, Verma DPS 1993 Proline biosynthesis and osmoregulation in plants. Plant Journal 4:215–223.

[CIT0014] Dennison KL, Robertson WR, Lewis BD 2001 Functions of AKT1 and AKT2 potassium channels determined by studies of single and double mutants of Arabidopsis. Plant Physiology 127:1012–1019. 11706182 PMC129271

[CIT0015] Du Y, Huang ZL 2008 Effects of seed mass and emergence time on seedling performance in *Castanopsis chinensis*. Forest Ecology Management 255:2495–2501.

[CIT0016] Ebstamp MJM, Vander Meerl M, Spronk BA 1994 Accumulation of fructose polymers in transgenic tobacco. Biotechnology 12:272–275. 7764488 10.1038/nbt0394-272

[CIT0017] Egilla JN, Davies FT, Drew LMC 2001 Effect of potassium on drought resistance of *Hibiscus rosasinensis* CV. Leprechaun: plant growth, leaf macto-and micronutrient content and root longevity. Plant and Soil 229:213–224.

[CIT0018] Genty B, Briantais JM, Baker NR 1989 The relationship between quantum yield of photosynthetic electron transport and quenching of chlorophyll fluorescence. Biochemica et Biophysica Acta 900:87–92.

[CIT0019] Hare PD, Cress WA, Staden JV 1998 Dissecting the roles of osmolyte accumulation during stress. Plant Cell Environment 21:535–553.

[CIT0020] Iqbal N, Ashraf Y, Ashraf M 2011 Modulation of endogenous levels of some key organic metabolites by exogenous application of glycine betaine in drought stressed plants of sunflower (*Helianthus annuus* L.). Plant Growth Regulation 63:7–12.

[CIT0021] Jander G, Joshi V 2010 Recent progress in deciphering the biosynthesis of aspartate-derived amino acids in plants. Molecular Plant 3:54–65. 20019093 10.1093/mp/ssp104

[CIT0022] Kato A, Tohoyama H, Joho M, Inouhe M 2007 Different effects of galactose and mannose on cell proliferation and intracellular soluble sugar levels in *Vigna angularis* suspension cultures. Journal of Plant Research 120:713–719. 17917698 10.1007/s10265-007-0117-9

[CIT0023] Kind T, Wohlgemuth G, Lee DY, Lu Y, Palazoglu M, Shahbaz S, Fiehn O 2009 Fiehnlib: mass spectral and retention index libraries for metabolomics based on quadrupole and time-of-flight gas chromatography/mass spectrometry. Analytical Chemistry 81:10038–10048. 19928838 10.1021/ac9019522 PMC2805091

[CIT0024] Kingsbury RW, Epstein E 1984 Selection for salt resistant in spring wheat. Crop Science 24:310–315.

[CIT0025] Kobayashi F, Takumi S, Nakata M, Ohno R, Nakamura T, Nakamura C 2004 Comparative study of the expression profiles of the Cor/Lea gene family in two wheat cultivars with contrasting levels of freezing tolerance. Physiologia Plantarum 120:585–594. 15032820 10.1111/j.0031-9317.2004.0293.x

[CIT0026] Koyro HW 2006 Effect of salinity on growth, photosynthesis, water relations and solute composition of the potential cash crop halophyte *Plantago coronopus* L. Environmental and Experimental Botany 56:136–146.

[CIT0027] Lehmann S, Funck D, Szabados L, Rentsch D 2010 Proline metabolism and transport in plant development. Amino Acids 39:949–962. 20204435 10.1007/s00726-010-0525-3

[CIT0028] Less H, Galili G 2008 Principal transcriptional programs regulating plant amino acid metabolism in response to abiotic stresses. Plant Physiology 147:316–330. 18375600 10.1104/pp.108.115733 PMC2330312

[CIT0029] Lisec J, Schauer N, Kopka J, Willmitzer L, Fernie AR 2006 Gas chromatography mass spectrometry-based metabolite profiling in plants. Nature Protocols 1:387–396. 17406261 10.1038/nprot.2006.59

[CIT0030] Loka DA, Oosterhuis DM 2014 Water-deficit stress effects on pistil biochemistry and leaf physiology in cotton (*Gossypium hirsutum*, L.). South African Journal of Botany 93:131–136.

[CIT0031] Loutfy N, El-Tayeb MA, Hassanen AM, Moustafa MF, Sakuma Y, Inouhe M 2012 Changes in the water status and osmotic solute contents in response to drought and salicylic acid treatments in four different cultivars of wheat (*Triticum aestivum*). Journal of Plant Research 125:173–184. 21445718 10.1007/s10265-011-0419-9

[CIT0032] Luis S, Pereira I, Cordery I, Iacovides L 2012 Improved indicators of water use performance and productivity for sustainable water conservation and saving. Agricultural Water Management 108:39–51.

[CIT0033] Marcin′ska I, Czyczyło-Mysza I, Skrzypek E, Filek M, Grzesiak S, Grzesiak MT, Janowiak F, Hura T, Dziurka M, Dziurka K, Nowakowska A, Quarrie SA 2013 Impact of osmotic stress on physiological and biochemical characteristics in drought-susceptible and drought-resistant wheat genotypes. Acta Physiology Plant 35:451–461.

[CIT0034] Meng J, Zhang X, Wu H, Bu J, Shi C, Deng C, Mao Y 2011 Morphine-induced conditioned place preference in mice: metabolomic profiling of brain tissue to find ‘molecular switch’ of drug abuse by gas chromatography/mass spectrometry. Analytica Chimica Acta 710:125–130. 22123121 10.1016/j.aca.2011.09.033

[CIT0035] Miller G, Suzuki N, Ciftci-Yilmaz S, Mittler R 2010 Reactive oxygen species homeostasis and signalling during drought and salinity stresses. Plant, Cell & Environment 33:453–467. 10.1111/j.1365-3040.2009.02041.x 19712065

[CIT0036] Oliver MJ, Guo LN, Alexander DC, Ryals JA, Wone BW 2011 A sister group contrast using untargeted global metabolomic analysis delineates the biochemical regulation underlying desiccation tolerance in *Sporobolus stapfianus*. Plant Cell 23:1231–1248. 21467579 10.1105/tpc.110.082800 PMC3101564

[CIT0037] Paz H, Martinez-Ramos M 2003 Seed mass and seedling performance within eight species of Psychotria (Rubiaceae). Ecology 84:439–450.

[CIT0038] Pettigrew WT 2004 Physiological consequences of moisture deficit stress in cotton. Crop Science 44:1265–1272.

[CIT0039] Plauborg F, Abrahamsen P, Gjettermann B, Mollerup M, Iversen BV, Liu FL, Andersen MN, Hansen S 2010 Modelling of root ABA synthesis, stomatal conductance, transpiration and potato production under water saving irrigation regimes. Agricultural Water Management 98:425–439.

[CIT0040] Renberg L, Johansson AI, Shutova T, Stenlund H, Aksmann A 2010 A metabolomic approach to study major metabolite changes during acclimation to limiting CO_2_ in *Chlamydomonas reinhardtii*. Plant Physiology 154:187–196. 20634393 10.1104/pp.110.157651 PMC2938146

[CIT0041] Rhodes D, Hanson AD 1993 Quaternary ammonium and tertiary sulphonium compounds in higher plants. Annual Review of Plant Biology 44:357–383.

[CIT0042] Ruan CJ, Teixeira da Silva JA 2011 Metabolomics: creating new potentials for unraveling the mechanisms in response to salt and drought stress and for the biotechnological improvement of xero-halophytes. Critical Reviews in Biotechnology 31:153–169. 21058928 10.3109/07388551.2010.505908

[CIT0043] Rzepka A, Rut G, Krupa J 2009 Effect of abiotic stress factors on fluctuations in contents of malate and citrate and on malic enzyme activity in moss gametophores. Photosynthetica 47:141–145.

[CIT0044] Santos MG, Pimentel C 2009 Daily balance of leaf sugars and amino acids as indicators of common bean (*Phaseolus vulgaris* L.) metabolic response and drought intensity. Physiology and Molecular Biology of Plants 15:23–30. 23572909 10.1007/s12298-009-0002-1 PMC3550377

[CIT0045] Schaneberg BT, Crockett S, Bedir E, Khan IA 2003 The role of chemical fingerprinting: application to *Ephedra*. Phytochemistry 62:911–918. 12590118 10.1016/s0031-9422(02)00716-1

[CIT0046] Shan FH, Tian LP, Zhang FT, Pang BS, Gao XH, Ren LP, Hou SM, Zhao CP 2012 Search of Jingdong series state trial wheat varieties selection and breeding. Journal of Agricultural Science 33:17−20.

[CIT0047] Shao HB, Chu LY, Wu G, Zhang JH, Lu ZH 2007 Where is the road to bio-water-saving for the globe?Colloids and Surfaces B: Biointerfaces 55:1–7. 17240122 10.1016/j.colsurfb.2006.12.001

[CIT0048] Shi DC, Zhao KF 1997 Effects of NaCl and Na_2_CO_3_ on growth of *Puccinellia tenuiflora* and on present state of mineral elements in nutrient solution. Acta Pratacuhurae Sinica 6:51–61.

[CIT0049] Shi YC 1999 To raise the water use efficiency by modern biotechnology. Science and Technology Review 2:1–5.

[CIT0050] Spickett CM, Smirnoff N, Ratdiffe RG 1992 Metabolic responses of maize roots to hyperosmotic ahock. Plant Physiology 99:856–863. 16669012 10.1104/pp.99.3.856 PMC1080556

[CIT0051] Stendle E, Peterson CA 1998 How does water get through roots?Journal of Experimental Botany 49:775–788.

[CIT0052] Sun JZ, Guo RJ, Zhang FS, Tian LP, Yin JY, Xue MS 2001 A summary on the ideas and strategy for selecting winter heat variety jingdong no.8. Journal of Laiyang Agmcultural College 18:274−279.

[CIT0053] Tambussi EA, Nogués S, Araus JL 2005 Ear of durum wheat under water stress: water relations and photosynthetic metabolism. Planta 221:446–458. 15645303 10.1007/s00425-004-1455-7

[CIT0054] Turk SCHJ, Smeekens S 1999 Genetic modification of plant carbohydrate metabolism. In: Chopra VL, Malik VS, Bhat SR, eds. Applied plant biotechnology. Science Publishers Inc, New Hampshire, USA, 71–100.

[CIT0055] Verdonk CJ, Vosch R, Verhoeven HA, Haring MA, Tunen AJV, Schuurink RC 2003 Regulation of floral scent production in petunia revealed by targeted metabolomics. Phytochemistry 62:997–1008. 12590126 10.1016/s0031-9422(02)00707-0

[CIT0056] Wang HX, Liu CM, Zhang L 2002 Water-saving agriculture in China: an overview. Advances in Agronomy 75:135–171.

[CIT0057] Wang H, Zhang M, Guo R, Shi D, Liu B, Lin X, Yang C 2012 Effects of salt stress on ion balance and nitrogen metabolism of old and young leaves in rice (*Oryza sativa* L.). BMC Plant Biology 12:194. 23082824 10.1186/1471-2229-12-194 PMC3496643

[CIT0058] Wei Y, Xu X, Tao H, Wang P 2006 Growth performance and physiological response in the halophyte *Lycium barbarum* grown at salt-affected soil. Annals of Applied Biology 149:263−269.

[CIT0059] Wenzel A, Frank T, Reichenberger G, Herz M, Engel KH 2015 Impact of induced drought stress on the metabolite profiles of barley grain. Metabolomics 11:454−467.

[CIT0060] Wu D, Shen Q, Cai S, Chen ZH, Dai F, Zhang G 2013 Ionomic responses and correlations between elements and metabolites under salt stress in wild and cultivated barley. Plant & Cell Physiology 54:1976–1988. 24058150 10.1093/pcp/pct134

[CIT0061] Xia J, Mandal R, Sinelnikov IV, Broadhurst D, Wishart DS 2012 Metaboanalyst 2.0—a comprehensive server for metabolomic data analysis. Nucleic Acids Research 40:W127–W133. 22553367 10.1093/nar/gks374 PMC3394314

[CIT0062] Yasar F, Uzal O, Tufenkci S, Yildiz K 2006 Ion accumulation in different organs of green bean genotypes grown under salt stress. Plant Soil Environment 52:476–480.

[CIT0063] Yang C, Chong J, Kim C, Li C, Shi D, Wang D 2007 Osmotic adjustment and ion balance traits of an alkaline resistant halophyte Kochia sieversiana during adaptation to saline and alkaline conditions. Plant Soil 294:263–276.

[CIT0064] Yang C, Jianaer A, Li C, Shi C, Wang D 2008 Comparison of the effects of salt-stress and alkaline-stress on photosynthesis and energy storage of an alkali-resistant halophyte *Chloris virgata*. Photosynthetica 46:273–278.

[CIT0065] Yang C, Xu HH, Wang L, Liu J, Shi DC, Wang D 2009 Comparative effects of salt-stress and alkaline-stress on the growth, photosynthesis, solute accumulation, and ion balance of barley plants. Photosynthetica 47:79–86.

[CIT0066] Yang XQ, Zhang SQ, Liang ZS, Shan Y 2004 Effects of water stress on chlorophyll fluorescence parameters of different drought resistance winter wheat cultivars seedlings. Acta Botanica Boreali-occidentalia Sinica 24:812−816.

[CIT0067] Zhang J, Zhang Y, Du Y, Chen S, Tang H 2011 Dynamic metabonomic responses of tobacco (*Nicotiana tabacum*) plants to salt stress. Journal of Proteome Research 10:1904–1914. 21323351 10.1021/pr101140n

[CIT0068] Zhao CP, Tian LP 2008 New varieties of high and stable yield winter wheat-JingDong 17. Journal of Triticeae Crops 28: 541−543.

[CIT0069] Zhao YJ, Weng BQ, Wang YX, Xu GZ 2009 Plant physio-ecological responses to drought stress and its research progress. Fujian Science and Technology of Rice and Wheat 27:45–50.

